# A bacteria-derived tail anchor localizes to peroxisomes in yeast and mammalian cells

**DOI:** 10.1038/s41598-018-34646-7

**Published:** 2018-11-06

**Authors:** Güleycan Lutfullahoğlu-Bal, Ayşe Bengisu Seferoğlu, Abdurrahman Keskin, Emel Akdoğan, Cory D. Dunn

**Affiliations:** 1 Institute of Biotechnology, Helsinki Institute of Life Science, University of Helsinki, 00014 Helsinki, Finland; 2 Department of Molecular Biology and Genetics, Koç University, 34450 Sarıyer, İstanbul Turkey; 3 Present Address: Department of Biological Sciences, Columbia University, New York, NY 10027, United States of America; 4 Present Address: Department of Microbiology and Molecular Genetics, University of California, Davis, Davis, CA 95616 United States of America

## Abstract

Prokaryotes can provide new genetic information to eukaryotes by horizontal gene transfer (HGT), and such transfers are likely to have been particularly consequential in the era of eukaryogenesis. Since eukaryotes are highly compartmentalized, it is worthwhile to consider the mechanisms by which newly transferred proteins might reach diverse organellar destinations. Toward this goal, we have focused our attention upon the behavior of bacteria-derived tail anchors (TAs) expressed in the eukaryote *Saccharomyces cerevisiae*. In this study, we report that a predicted membrane-associated domain of the *Escherichia coli* YgiM protein is specifically trafficked to peroxisomes in budding yeast, can be found at a pre-peroxisomal compartment (PPC) upon disruption of peroxisomal biogenesis, and can functionally replace an endogenous, peroxisome-directed TA. Furthermore, the YgiM(TA) can localize to peroxisomes in mammalian cells. Since the YgiM(TA) plays no endogenous role in peroxisomal function or assembly, this domain is likely to serve as an excellent tool allowing further illumination of the mechanisms by which TAs can travel to peroxisomes. Moreover, our findings emphasize the ease with which bacteria-derived sequences might target to organelles in eukaryotic cells following HGT, and we discuss the importance of flexible recognition of organelle targeting information during and after eukaryogenesis.

## Introduction

While prokaryotes can harbor compartments dedicated to specific functions and biochemical reactions^1^, eukaryotes are commonly characterized by a higher level of compartmentalization by membranous structures. One of these organelles, the peroxisome, is bounded by a single membrane and is often a location of fatty acid oxidation in eukaryotic cells^2,3^. Beyond fatty acid breakdown, peroxisomes play multiple roles among eukaryotes^4,5^, including sterol synthesis^6^, synthesis of ether lipids^7^, and even glycolysis^8^. Soluble proteins are directed to the lumen, or matrix, of peroxisomes by a conserved import machinery commonly (but not exclusively) taking advantage of a carboxyl-terminal sequence called peroxisomal targeting sequence 1 (PTS1)^9,10^. Membrane proteins are also targeted to peroxisomes, but mechanisms of peroxisomal membrane protein (PMP) biogenesis are not as well characterized as those processes that mediate import to the peroxisomal matrix^11,12^. The evolutionary origin of peroxisomes is obscure, although some evidence suggests that the core machinery required for peroxisomal assembly is derived from the endoplasmic-reticulum-associated protein degradation (ERAD) machinery^13,14^.

During and following eukaryogenesis, (proto-)nuclear genes were obtained by gene transfers from endosymbionts and from free-living prokaryotes, with some of these proteins subsequently targeted to organelles^15–20^. Beyond more ‘ancient’ gene transfers, HGT from prokaryotes to eukaryotes and conversion of endosymbionts to organelles appears to continue at present day^21–24^. Signals found within the polypeptide sequence of nucleus-encoded genes play a dominant role in targeting to eukaryotic organelles, and how prokaryote-derived proteins might acquire such sequences and become localized to eukaryotic organelles is a topic of intense inquiry. In a previous study directed toward the principals of organelle targeting following HGT from bacteria^25^, we focused our attention upon those proteins predicted to be anchored to membranes by a carboxyl-terminal hydrophobic stretch of amino acids, or tail anchor (TA). Here, we describe the trafficking of one of these bacteria-derived TAs, retrieved from the YgiM protein of *E. coli*. We find that the YgiM tail anchor sequence [YgiM(TA)] localizes to peroxisomes in yeast and in human cells and appears to functionally replace an endogenous, peroxisomal TA in *S. cerevisiae*. In mutants for which peroxisomal biogenesis is impaired, the YgiM(TA) is localized to ER or to ER-derived pre-peroxisomal compartments (PPCs), suggesting that this exogenous domain follows a trafficking pathway used by endogenously encoded peroxisomal TAs. Our work highlights the ability of eukaryotes to use prokaryotic information obtained by HGT to direct acquired proteins to distinct subcellular locations.

## Results

### A domain encoded by the bacterial YgiM gene is targeted to the peroxisomes of yeast cells

**Figure 1:**
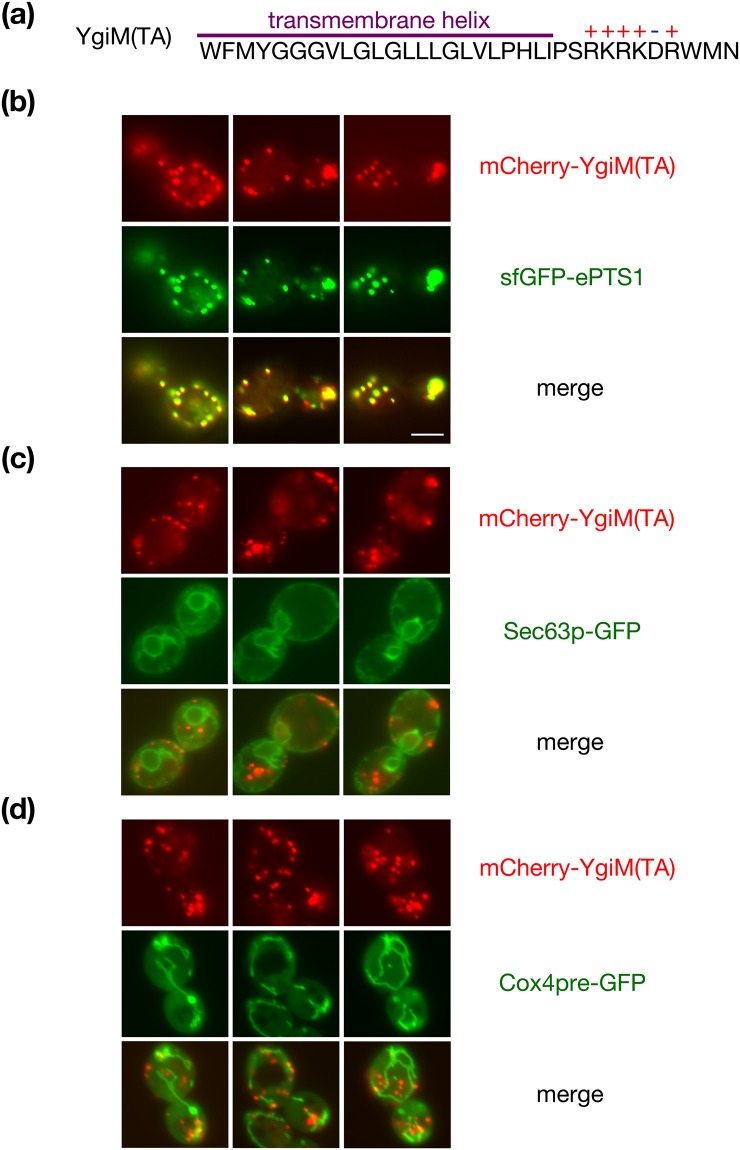
The predicted tail anchor of *Escherichia coli* YgiM localizes to peroxisomes in *Saccharomyces cerevisiae*. (**a**) The sequence of the YgiM(TA), as retrieved from UniProt^76^ record P0ADT8, is provided. The transmembrane helix is predicted using the TMHMM 2.0 server^77^ and charged residues are indicated. (**b**) The YgiM(TA) co-localizes with a protein targeted to peroxisomes. Strain BY4741, harboring plasmid b311 (sfGFP-ePTS1), was mated to strain BY4742, carrying plasmid b274 [mCherry-YgiM(TA)]. The resulting diploids were analyzed by fluorescence microscopy of live cells. (**c**) The YgiM(TA) does not co-localize with ER in wild-type cells. Strain BY4741, containing plasmid pJK59 (Sec63p-GFP) and strain BY4742, carrying plasmid b274 [mCherry-YgiM(TA)] were analyzed as in (**b**). (**d**) The YgiM(TA) does not co-localize with mitochondria. Strains BY4741 and BY4742, transformed with plasmids pHS1 (Cox4pre-GFP) and b274 [mCherry-YgiM(TA)], respectively, were mated and analyzed as in (**b**).

During a previous appraisal of the ability of eukaryotic cells to utilize potential targeting information encoded by prokaryotes^25^, we fused mCherry to the amino-terminus of predicted TAs encoded by the *E. coli* genome. These fluorescent fusion proteins were found at diverse locations within the cell, and we noted that mCherry fused to amino acids 173–206 of the uncharacterized YgiM protein, hereafter entitled the YgiM(TA), was found in a punctate pattern reminiscent of peroxisomes. The YgiM(TA) contains a predicted transmembrane helix followed by a positively charged lumenal tail (Fig. 1a). In order to determine whether the YgiM(TA) might indeed target to peroxisomes, we expressed mCherry-YgiM(TA) from the strong *ADH1* promoter together with superfolder green fluorescent protein (sfGFP) linked to the enhanced peroxisomal targeting signal 1 (ePTS1)^26^. mCherry-YgiM(TA) co-localized with sfGFP-ePTS1, providing strong evidence of YgiM(TA) targeting to peroxisomes (Fig. 1b). In contrast, mCherry-YgiM(TA) was not detectable at the endoplasmic reticulum (ER) (Fig. 1c). Similarly, mCherry-YgiM(TA) was not detectable at mitochondria (Fig. 1d), even upon deletion of Msp1p (Supplementary Fig. S1), which extracts peroxisomal tail-anchored proteins mistargeted to mitochondria^27,28^.

### The YgiM tail anchor can functionally replace an endogenous, peroxisome-directed tail anchor

Pex15p, which participates in the import of yeast proteins to the peroxisomal matrix^9,10,29^, is the only *S. cerevisiae* protein thought to be directed specifically to peroxisomes by a TA^30^. A lack of Pex15p at peroxisomes leads to defective peroxisomal biogenesis and cytosolic accumulation of PTS1-directed proteins^31^. Previous studies have demonstrated that Pex15p is functional when its TA is replaced by that of the mammalian PEX26 protein^32^, suggesting that other peroxisome-inserted TAs might also support Pex15p activity. Therefore, we tested whether the YgiM(TA) might target the Pex15p cytosolic domain to peroxisomes and permit Pex15p-driven protein import.

**Figure 2:**
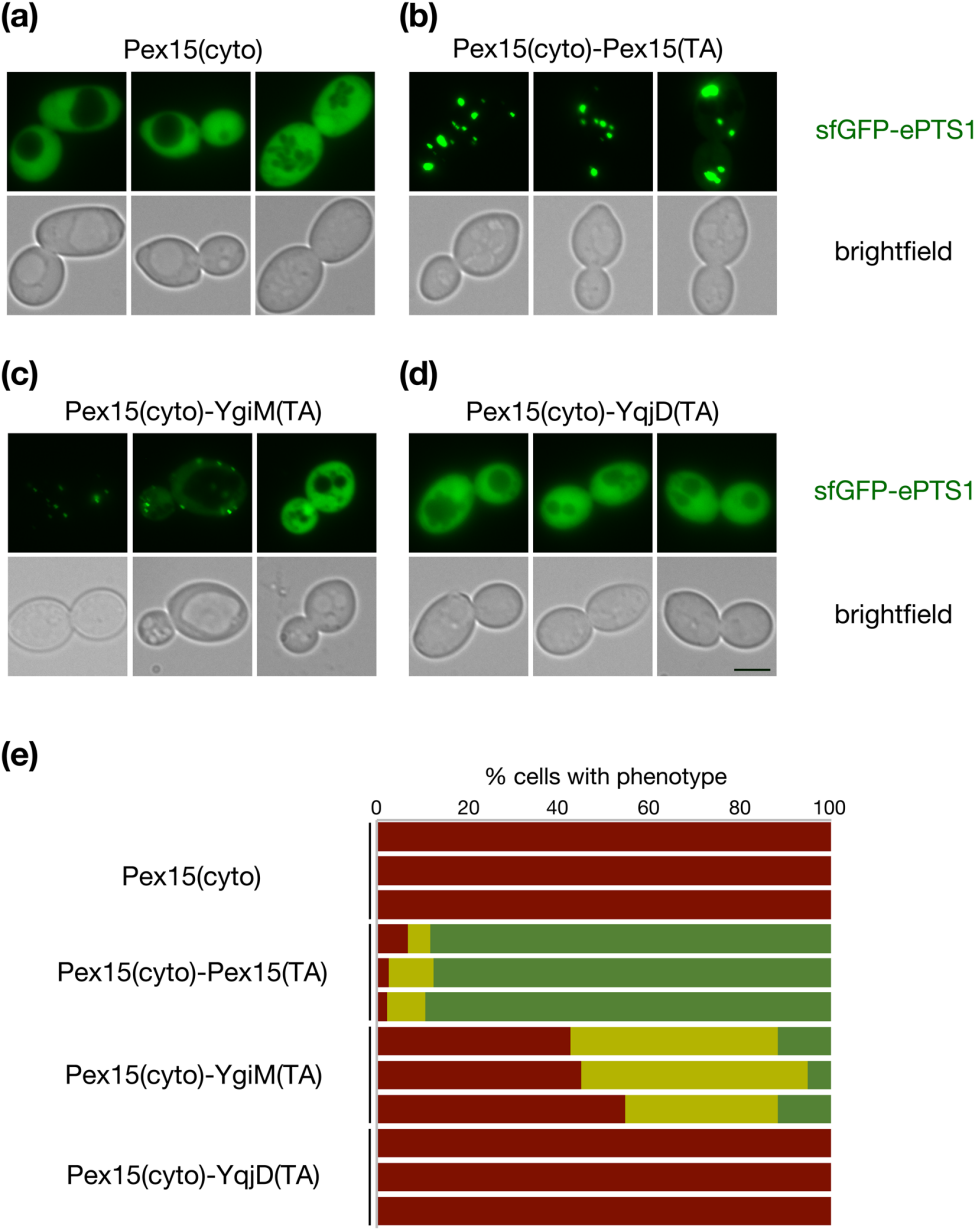
The YgiM(TA) can functionally replace the tail anchor of Pex15p. *pex15∆/pex15∆* strain CDD1182, containing a counter-selectable plasmid expressing the Pex15p cytosolic domain (cyto) fused to its own TA (b354) was transformed with plasmids expressing (**a**) Pex15(cyto) lacking a TA (b341) (**b**) Pex15(cyto)-Pex15(TA) (b326) (**c**) Pex15(cyto) fused to the YgiM(TA) (b329) or (**d**) Pex15(cyto) fused to the YqjD(TA) (b330). Plasmid b354 was then removed by counter-selection with CHX, and Pex15p function was assessed by sfGFP-ePTS1 localization to puncta indicative of peroxisomes competent for import of matrix-directed proteins. (**e**) Reports the quantification, blinded to genotype during analysis, of three independent experiments. Red represents cells with diffuse signal in the nucleus and cytosol but no puncta, yellow represents cells with both diffuse and punctate signal, and green represents cells in which only punctate signal could be detected (n > 200 cells per sample in each experimental replicate).

As expected, expression of an untethered Pex15p cytosolic domain (amino acids 1–331)^32^ under control of the native *PEX15* promoter in cells lacking a chromosomal copy of *PEX15* did not allow localization of sfGFP-ePTS1 to puncta (Fig. 2a,e), while re-attachment of the Pex15(TA) to the Pex15p cytosolic domain permitted sfGFP-ePTS1 recruitment to puncta suggestive of import into the peroxisomal matrix (Fig. 2b,e). Indicating that the bacterial YgiM(TA) can provide functionality in *S. cerevisiae*, Pex15(1–331)-YgiM(TA) allowed punctate localization of sfGFP-ePTS1, although rescue of the *pex15∆* phenotype was not absolute (Fig. 2c,e). Not all bacteria-derived TAs seem to support Pex15p function: Pex15(1–331) fused to the *E. coli* YqjD(TA), which was previously demonstrated^25^ to target predominantly to mitochondria and, to a lesser extent, the ER, failed to permit recruitment of sfGFP-ePTS1 to puncta in *pex15∆* cells (Fig. 2d,e). Though a portion of the *S. cerevisiae* Fis1p is associated with peroxisomes^33^, we found no evidence that the Fis1p(TA) can allow Pex15p function (Supplementary Fig. S2).

### The YgiM tail anchor resides within a pre-peroxisomal compartment upon disruption of peroxisomal biogenesis

In yeast, many integral peroxisomal membrane proteins (PMPs) are inserted at the ER before subsequent trafficking to peroxisomes^12^. The tail-anchored Pex15 protein is also thought to begin its journey to the peroxisome within the ER^30,34^, and upon disruption of PMP trafficking, accumulates in ER-derived pre-peroxisomal compartments (PPCs)^35^ marked by Pex14p^36–38^, a component contributing to formation of the mature peroxisomal import pore^39,40^. To visualize Pex14p-marked PPCs, we tagged endogenous Pex14p with sfGFP. The Pex14p-sfGFP fusion protein was easily detectable, could promote peroxisomal protein import (Supplementary Fig. S3), and continued to be localized, as previously reported, in puncta representing PPCs upon disruption of peroxisomal biogenesis by removal of Pex3p or Pex19p.

We tested whether the Pex15(TA) would associate with PPCs. The Pex15(TA) with mCherry fused to its amino-terminus was indeed found to co-localize with Pex14p at peroxisomes of wild-type (WT) cells (Supplementary Fig. S4), although a notable fraction of mCherry-Pex15(TA) is also mistargeted to mitochondria. Consistent with a previous report examining the trafficking of full-length Pex15p^35^, we found that mCherry-Pex15(TA) could be co-localized with Pex14p-sfGFP upon disruption of PMP trafficking by removal of Pex3p or Pex19p.

**Figure 3:**
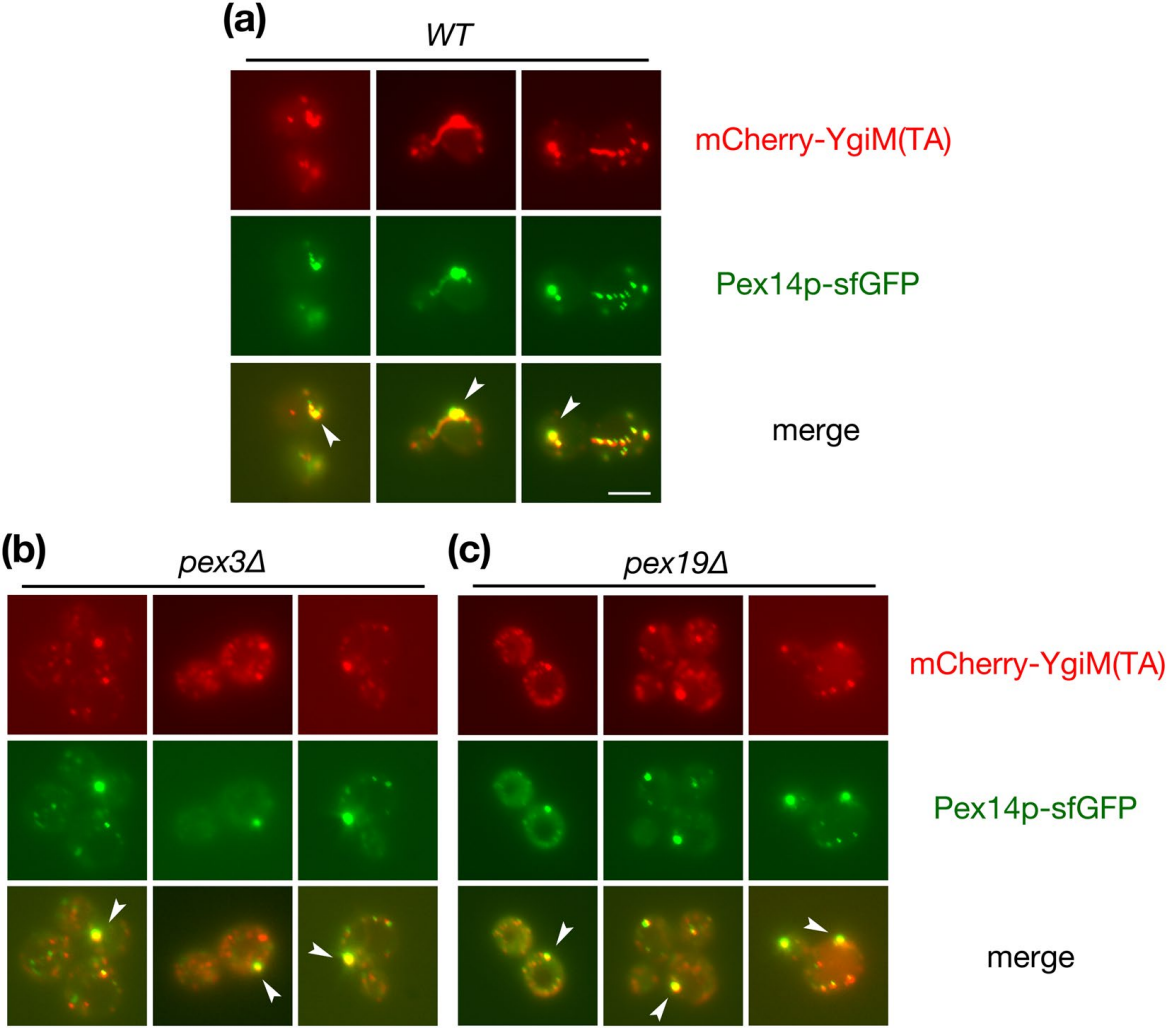
The YgiM(TA) is associated with PPCs containing Pex14p upon blockade of PMP transit to peroxisomes. (**a**) WT (CDD1200) (**b**) *pex3∆* (CDD1201) or (**c**) *pex19∆* (CDD1202) isolates expressing mCherry-YgiM(TA) from plasmid b274 and Pex14p-sfGFP from the native *PEX14* locus were examined by fluorescence microscopy of live cells. White arrows provide examples of locations at which Pex14p-sfGFP resides near mCherry-YgiM(TA).

If YgiM(TA) is, like endogenous PMPs, initially targeted to ER, this domain might similarly be localized to PPCs upon disruption of PMP trafficking. mCherry-YgiM(TA) co-localized with Pex14p-sfGFP at mature peroxisomes in wild-type cells (Fig. 3a), and indeed, mCherry-YgiM(TA) continued to co-localize with Pex14p-sfGFP in *pex3∆* (Fig. 3b) or *pex19∆* (Fig. 3c) cells. Our findings are consistent with trafficking of the YgiM(TA) to the ER, then to PPCs, before subsequent movement to peroxisomes, and our results suggest consonance between cellular pathways handing the endogenous Pex15(TA) and the bacterial, exogenous YgiM(TA).

### The ER-localized Spf1 protein contributes to the trafficking of the YgiM tail anchor to peroxisomes

**Figure 4:**
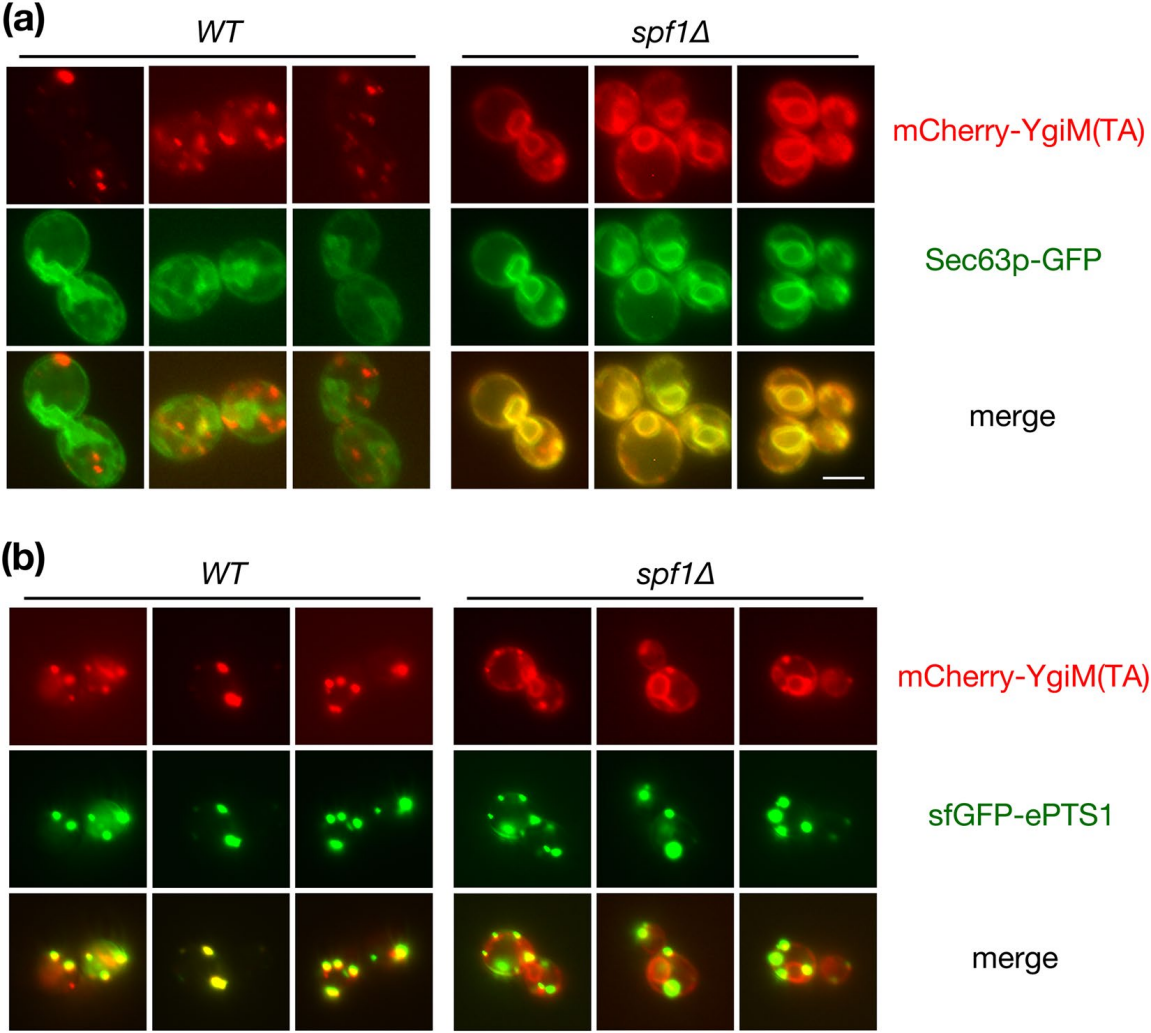
The YgiM(TA) is mislocalized to the endoplasmic reticulum upon deletion of the Spf1 protein. WT (BY4742) or *spf1∆* (CDD949) cells expressing mCherry-YgiM(TA) from plasmid b274 and either (**a**) Sec63p-GFP from plasmid pJK59 or (**b**) sfGFP-ePTS1 from plasmid b311 were examined by live-cell fluorescence microscopy.

Spf1p, an ER-localized protein involved in manganese transport^41^, plays a role in peroxisomal biogenesis^42,43^, and the localization of at least two proteins capable of trafficking from ER to peroxisomes, Pex3p and Ant1p^44–47^, is altered by Spf1p removal^48^. Consequently, we investigated whether trafficking of YgiM(TA), like endogenously encoded PMPs, might be affected by Spf1p deletion. Indeed, mCherry-YgiM(TA) was significantly redistributed to ER in *spf1∆* cells (Fig. 4), demonstrating a potential role for Spf1p in the targeting of peroxisome-directed TAs and consistent with initial YgiM(TA) movement through the ER. Interestingly, some mCherry-YgiM(TA) could also be found at mature peroxisomes marked by sfGFP-ePTS1 in *spf1∆* cells, demonstrating that Spf1p removal does not completely abolish TA trafficking. We also note that Spf1p is apparently not required for the generation of PPCs containing Pex14p, since Pex14p-sfGFP puncta are easily visualized in *spf1∆* cells, including within cells also deleted of Pex3p or Pex19p (G Lutfullahoğlu-Bal, unpublished data).

We tested whether a construct anchored by the TA of the endogenous Pex15p would, like the YgiM(TA), be found predominantly at the ER upon deletion of Spf1p. Indeed, like mCherry-YgiM(TA), mCherry-Pex15(TA) was localized abundantly to ER in *spf1∆* cells but not in WT cells (Supplementary Fig. S5), again suggesting congruence between trafficking mechanisms used by the exogenous YgiM(TA) and the endogenous Pex15(TA).

### Expression of the YgiM(TA) does not disturb peroxisomal biogenesis

**Figure 5:**
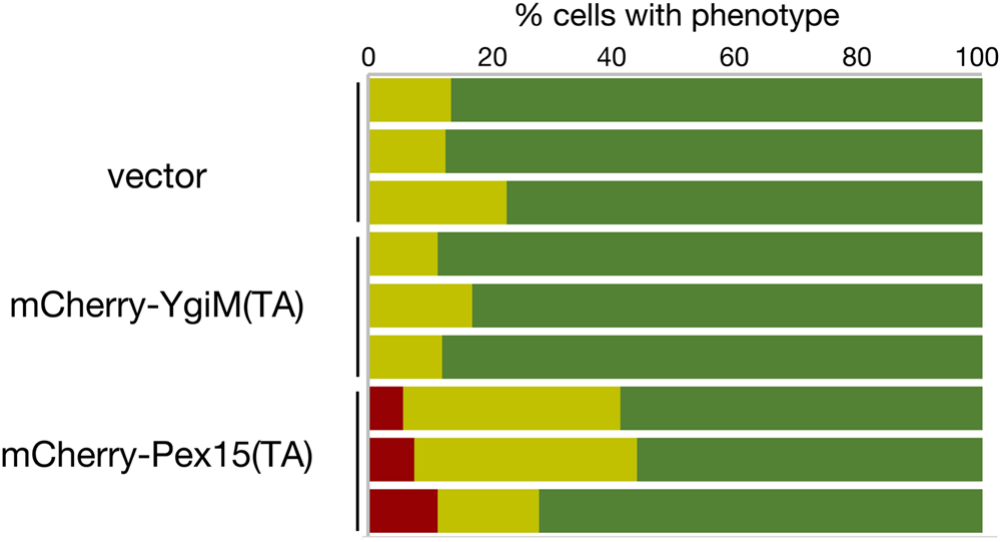
Expression of the YgiM(TA) does not perturb peroxisomal biogenesis. WT cells (BY4742) expressing sfGFP-ePTS1 from plasmid b311 along with mCherry-YgiM(TA) from plasmid b274, mCherry-Pex15(TA) from plasmid b365, or empty vector pRS315 were examined as in Fig. 2e.

Successful study of a biological process often requires that the system under investigation is not perturbed by the chosen experimental approach. Since overexpression of full-length Pex15p, the only endogenous, peroxisome-specific TA, is known to perturb peroxisomal biogenesis^30^, we asked whether expression of only the TA domains of YgiM or Pex15p, driven by the strong *ADH1* promoter, would have a detrimental effect on peroxisome assembly. Toward this goal, the behavior of sfGFP-ePTS1 was assessed in cells expressing mCherry-YgiM(TA) or mCherry-Pex15(TA). Peroxisomal biogenesis was indeed disrupted by mCherry-Pex15(TA) overexpression (Fig. 5), with an average of 8% of cells lacking discernable peroxisomes across three independent experiments. Moreover, partial nucleocytoplasmic accumulation of sfGFP-ePTS1 was visible in nearly twice as many cells expressing mCherry-Pex15(TA) as those expressing empty vector. However, mCherry-YgiM(TA) expression had no effect on sfGFP-ePTS1 localization when compared to cells harboring empty vector; distinct peroxisomes could be visualized in all cells. Therefore, overexpression of the YgiM(TA), unlike overexpression of an endogenous peroxisome-directed TA, does not appear to readily disrupt peroxisomal biogenesis.

### The YgiM(TA) is localized to the peroxisomes of mammalian cells

**Figure 6:**
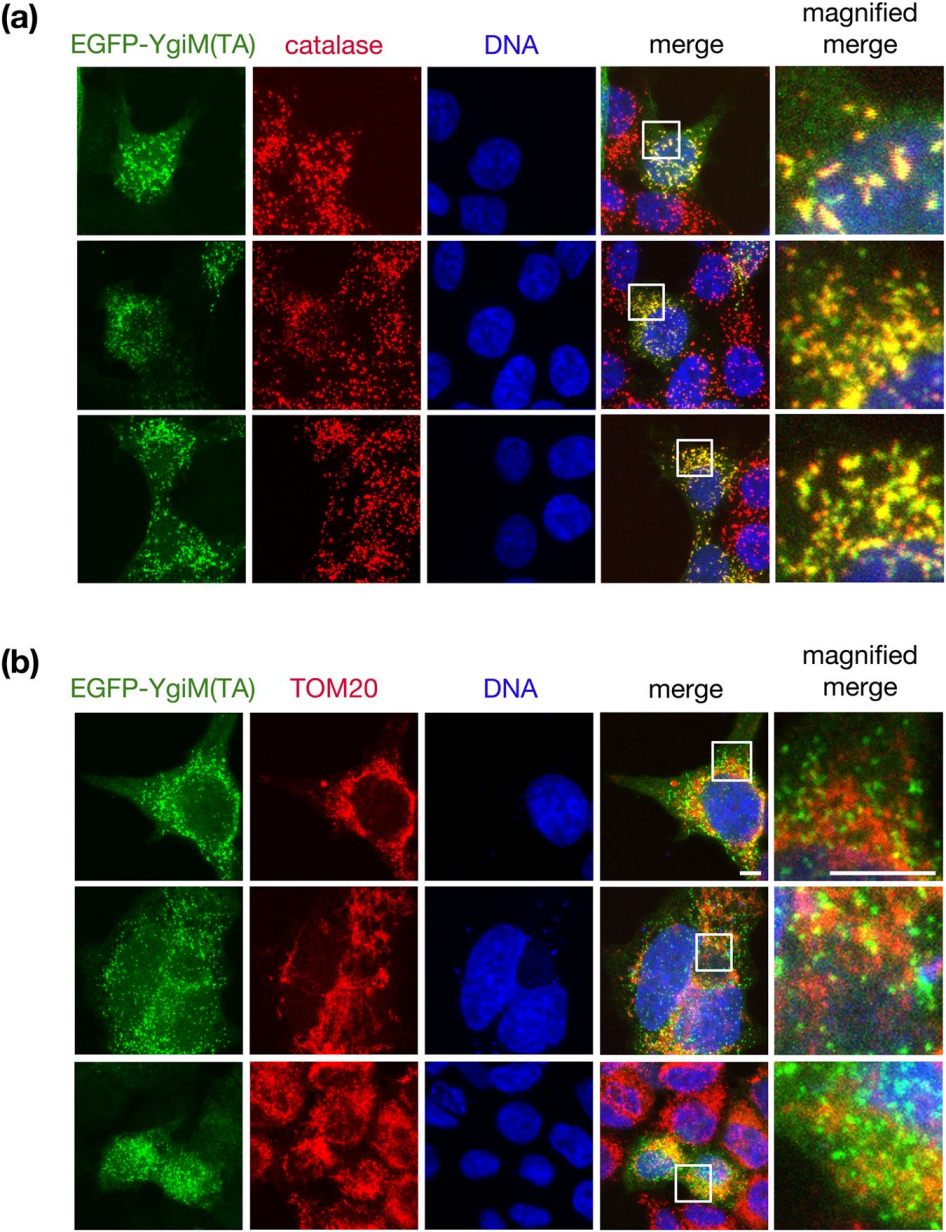
The YgiM(TA) localizes to peroxisomes in mammalian cells. Plasmid b374, expressing EGFP-YgiM(TA), was transiently transfected into HEK293T cells, and cultures were processed for immunofluorescence. Anti-GFP antibodies were used to detect EGFP-YgiM(TA), and DAPI was used to stain cellular DNA. (**a**) Anti-catalase antibodies were used to label mature peroxisomes, or (**b**) anti-TOM20 antibodies were used to label mitochondria. A white box defines the region of the image magnified in the right-most panel set.

Finally, we investigated whether YgiM(TA) might target to peroxisomes in mammalian cells, since the mechanism by which tail-anchored proteins are delivered to peroxisomes may differ between yeast and mammals^9,12,49^. Upon transient transfection of a construct in which enhanced green fluorescent protein (EGFP) is fused to the YgiM(TA), punctate structures suggesting peroxisomal localization were visualized in HEK293T cells (Fig. 6a). This localization was confirmed by co-localization with catalase, a marker of mature peroxisomes. As in yeast, EGFP-YgiM(TA) was not trafficked to mitochondria, as revealed by scant co-localization between EGFP-YgiM(TA) and the mitochondrial TOM20 protein (Fig. 6b).

## Discussion

### What features of YgiM(TA) allow targeting to peroxisomes?

The *E. coli* YgiM(TA) was detected at peroxisomes, with no microscopic or functional evidence of mitochondrial localization in yeast or in human cells. Conversely, TAs found within two other proteins encoded by the same organism, YqjD and ElaB, targeted to mitochondria and ER, with no evidence of peroxisomal localization^25^ (and this study). Other tail-anchored proteins can target to both mitochondria and peroxisomes. For example, human FIS1^50,51^, yeast Fis1p^33^, and human MFF are localized to both organelles^52^. The parameters that allow TAs to discriminate between peroxisomes, mitochondria, and other organelles are not understood, but may be related to the hydrophobicity of the membrane associated domain, along with the number and specific location of charges within the TA^53,54^. When considering the recent development of a classifier for peroxisome-directed mammalian TAs^54^, the GRAVY hydrophobicity score (1.7)^55^ of the YgiM transmembrane domain, denoting more limited hydrophobicity, together with the net charge (+4.1) of the proposed lumenal tail at neutral pH (Protein Calculator v3.4, http://protcalc.sourceforge.net/) do, in fact, predict peroxisomal localization of the YgiM(TA). We note that the biogenesis of YgiM in bacteria has not been investigated, and indeed full-length YgiM contains a predicted signal sequence at its amino-terminus^56^, indicating co-translational insertion and suggesting that any predicted TA would not drive initial membrane targeting in *E. coli*.

### Heterologous expression of YgiM(TA) may reveal mechanisms of tail-anchor trafficking to peroxisomes

Only two peroxisome-directed proteins harboring TAs are encoded by *S. cerevisiae*: Pex15p and Fis1p. Both associate with other peroxisomal proteins: Pex15p is found in a complex with Pex3p^57^, and Fis1p cooperates with Pex11p^58^. Based on these findings, one might propose a scenario in which both tail-anchored polypeptides obtain their final peroxisomal location solely through their functional assembly with other proteins not harboring a TA, and that no pathway with a specific role in directing tail-anchored client proteins to peroxisomes exists in budding yeast. However, the YgiM(TA), separated from eukaryotes by billions of years of evolutionary distance, appears to localize specifically to peroxisomes in both yeast and human cells. This exogenously expressed domain would not bind to any endogenous interaction partners in order to carry out a cellular function, yet makes its way to peroxisomes nonetheless, supporting the presence of a more generalized mechanism that allows trafficking of tail-anchored proteins to peroxisomes in *S. cerevisiae*.

Importantly, expression of native proteins at incorrect stoichiometry can perturb cellular functions that may be under investigation^59,60^, as illustrated by disruption of peroxisomal biogenesis upon overexpression of full-length Pex15p^30^. In this study, we found that the TA of Pex15p could also disrupt peroxisomal protein import, attenuating its value as an experimental substrate for studies of TA targeting in yeast. In contrast, YgiM(TA) expression did not affect peroxisomal assembly, and unlike overexpressed Pex15(TA), appears specifically targeted to peroxisomes. Moreover, the YgiM(TA) is also relatively short when compared to several other peroxisome-directed TAs, providing the opportunity for facile mutational analyses of YgiM(TA). Therefore, we suggest that heterologously expressed YgiM(TA) is likely to be a preferred substrate for further exploration of the mechanisms by which TAs reach peroxisomes.

### Why is protein targeting to eukaryotic organelles so permissive?

In this study, we have demonstrated that a predicted membrane insertion sequence obtained from a prokaryote can be directed to the peroxisomes of eukaryotic cells. Our findings expand upon earlier studies in which protein sequence derived from prokaryotes could traffic to eukaryotic organelles, such as ER and mitochondria^25,61–65^. We propose that the ability to direct prokaryotic protein sequences to eukaryotic organelles, even though these regions were not previously selected for targeting prowess in eukaryotes, might have been of general benefit to eukaryotes over evolutionary time. Specifically, failure to allow degeneracy among organelle targeting sequences^66^ would potentially have limited the utility of genes acquired from the proto-mitochondrial endosymbiont or from neighboring microorganisms near the dawn of eukaryogenesis, potentially slowing or forbidding the emergence of the eukaryotic cell^67^. In addition, the ability of eukaryotes to take advantage of genes acquired by HGT at present day^23,68,69^ could similarly be hampered by a strict sequence requirement, rather than lax structural requirements, for recognition of organelle-targeting signals contained within polypeptides. Additionally, sequestration at an organelle might avoid detrimental effects of aggregation or chaperone sequestration, and thereby avoid selection against an otherwise advantageous gene transfer, and this aspect of organelle targeting may be particularly important when considering hydrophobic regions like the TA examined in this study.

Encompassing the specific use of HGT-acquired genetic information would be a more general need for eukaryotic cells to harbor permissive organelle translocation machineries that allow recognition of degenerate import signals. Given that organelles are maintained in order to compartmentalize biochemical pathways and other cellular activities, it follows that multiple polypeptides will often act together as a module within a given organelle. Strict sequence requirements for organelle import would make it highly improbable that multiple proteins once cooperating at one cellular location, such as the cytosol, could later find themselves simultaneously localized together in a different cellular compartment. Conversely, more relaxed structural determinants of organelle-targeting regions of a protein that might be recognized by permissive substrate receptors, such as hydrophobicity and charge, would allow proteins already encoded by a cell to sample novel compartments. Eventually, as previously proposed by Martin^70^, organelle sampling by polypeptides, followed by further mutational tinkering with organelle targeting sequences, could lead to increased fitness through the localization of an entire cellular pathway to a new location. Moreover, genes can evolve *de novo*^71^, and the ability of newly generated polypeptides to test different organelle environments may also contribute to fitness or the exploration of a new ecological niche. Ultimately, then, the question of how the protein translocation machineries of organelles recognize targeting information of client proteins, obtained by HGT or as the outcome of other genetic processes, becomes a question of ‘evolvability’, or the advantageous capacity of a pedigree of organisms to more easily sample genotypic and phenotypic space^72^.

## Methodology

### Yeast strains, plasmids, and culture conditions

Culture conditions are as described in^73^. All experiments with *S. cerevisiae* have been performed at 30 °C. Plasmids, strains, and oligonucleotides used in this study can be located in the Supplementary Dataset.

### Assessment of Pex15 functionality

Diploid strain CDD1182, deleted of chromosomal *PEX15*, expressing peroxisome-targeted sfGFP from plasmid b311, and carrying a fully-functional fusion between the cytosolic and TA domains of Pex15p from plasmid b354 driven by the *PEX15* promoter, was transformed with plasmids expressing variants of Pex15p in which the cytosolic domain was fused to test TAs by a linker region consisting of Fis1p amino acids 119–128, a stretch of amino acids not necessary or sufficient for organelle targeting^74,75^. Strains were cultured overnight in supplemented minimal medium (SMM) lacking uracil and histidine (-Ura-His). Cells were then transferred to SMM-Ura-His containing 3 mg/L cycloheximide (CHX) and cultured overnight before fluorescence microscopy in the logarithmic phase of proliferation. Counter-selection of plasmid b354 was confirmed by lack of proliferation on medium lacking tryptophan.

### Mammalian cell culture and transfection

Cells were maintained at 37 °C and 5% CO_2_ and cultured in Dulbecco’s Modified Eagle’s Medium (DMEM) supplemented with 10% fetal bovine serum, 2 mM L-glutamine, 100 U/ml penicillin/streptomycin, and 50 μg/ml uridine. HEK293T cells were plated overnight before transfection in 500 μl of complete growth medium at a cell density of 1 × 10^5^ cells/ml in a 24-well plate containing glass coverslips. Transfection was performed using TransIT-2020 (Mirus Bio) reagent, and transfection mixture contained: 250 ng of plasmid b374, 50 μl of cell culture medium, and 1 μl of transfection reagent. The mixture was incubated at room temperature for 20 min, and transfection mixture was added drop-wise to the cells. Cells were fixed for immunofluorescence analysis 24 hr after transfection.

### Microscopy

Microscopy on yeast cells was performed using logarithmic phase cultures. Live-cell epifluorescence microscopy was performed using either a Nikon Eclipse 80i microscope equipped with a 100X Plan Fluor objective and DS-Qi1Mc camera or a Zeiss Axio Imager.M2 fixed with a 63X Plan-Apochromat/1.40 Oil DIC objective and AxioCam HR R3 camera. mCherry fusions are driven by the *ADH1* promoter and contain Fis1p amino acids 119–128 linking mCherry to each carboxyl-terminal organelle targeting sequence.

To carry out indirect immunofluorescence experiments on mammalian cells, transfected HEK293T cells were fixed using 4% paraformaldehyde in phosphate-buffered saline (PBS), pH 7.4 for 10 min at room temperature. Cells were washed three times with PBS for 5 min, then blocked for 1 hr using PBS containing 0.3% Triton X and 1% bovine serum albumin. Cells were then incubated overnight in primary antibodies (listed in Supplementary Dataset) diluted in blocking solution at 4 °C. Next, cells were washed 3x with PBS and incubated with secondary antibodies in the blocking solution for 1 h in the dark. After secondary antibody incubation, 4′,6-diamidino-2-phenylindole (DAPI) was added to a final concentration of 1 μg/ml for 10 min. Cells were again washed 3x with PBS, and coverslips were mounted using 80% glycerol prepared in 20 mM Tris-HCl pH 8.0. Coverslips were sealed and stored at 4 °C before microscopy. Imaging was performed using a Zeiss LSM700 Axio Imager.M2 confocal microscope equipped with an LCI Plan-Neofluar 63x/1.30 Imm Corr objective. Scale bars provided with yeast and mammalian cell microscopy images correspond to 5 µm.

## Electronic supplementary material

Supplementary Information

Supplementary Dataset

## Data Availability

The datasets generated and/or analysed during the current study are available from the corresponding author upon reasonable request.
