# Supplementary material for: A bacteria-derived tail anchor localizes to peroxisomes in yeast and mammalian cells
Source: Sci Rep. 2018 Nov 6;8:16374. doi: 10.1038/s41598-018-34646-7 (PMC6219538; doi:10.1038/s41598-018-34646-7)
Supplement: Supplementary file 1 — Supplementary Information [file 41598_2018_34646_MOESM1_ESM.pdf]

A bacteria-derived tail anchor localizes to peroxisomes in yeast and mammalian cells

Güleycan Lutfullahoğlu-Bal[a,b], Ayşe Bengisu Seferoğlu[a], Abdurrahman Keskin[b†], Emel Akdoğan[b‡], and Cory D. Dunn[a,b*]

SUPPLEMENTAL INFORMATION

SUPPORTING INFORMATION

Figure S1. The YgiM(TA) does not accumulate at mitochondria upon deletion of Msp1p. mCherry-YgiM(TA), encoded by plasmid b274 was expressed in *msp1Δ* (CDD1154) cells. (**a**) Peroxisomes were labelled with sfGFP-ePTS1 expressed from plasmid b311, or (**b**) mitochondria were labelled by Cox4pre-GFP expressed from plasmid pHS1, and live cells were examined by fluorescence microscopy.

Figure S2. The Pex15 cytosolic domain fused to the Fis1p TA is not functional. (**a**) *pex15Δ/pex15Δ* strain CDD1182 was transformed with plasmids expressing the Pex15p cytosolic domain (cyto) fused to the Fis1p(TA) (b327) or with empty vector pRS316, and plasmid b354 was removed by counter-selection. sfGFP-ePTS1 expressed from plasmid b311 was examined as in Figure 2e. Representative images are provided in (**b**).

Figure S3. The Pex14p-sfGFP fusion protein is functional and detectable in mutants in which PMP trafficking is blocked. WT (CDD1200), *pex3Δ* (CDD1201), and *pex19Δ* (CDD1202) strains, all harboring a chromosomal *PEX14-sfGFP* allele, were transformed with plasmid b364 expressing mCherry-ePTS1 and examined by live-cell microscopy.

Figure S4. The Pex15(TA) can be found at Pex14p-positive PPCs upon disruption of PMP trafficking to peroxisomes. (**a**) WT (CDD1200) (**b**) *pex3Δ* (CDD1201) or (**c**) *pex19Δ* (CDD1202) cells expressing mCherry-Pex15(TA) from plasmid b365 were examined as in Figure 3. White arrows provide examples of locations at which Pex14p-sfGFP resides near mCherry-Pex15(TA). (**d**) The Pex15(TA) is partially mislocalized to mitochondria in wild-type cells**.** Strain BY4741 was transformed with plasmid b365, expressing mCherry-Pex15(TA), and with plasmid pHS1, expressing Cox4pre-GFP. Live cells were examined by fluorescence microscopy.

Figure S5. The Pex15(TA) is concentrated at the endoplasmic reticulum upon deletion of the Spf1 protein. WT (BY4742) or *spf1Δ* (CDD949) cells expressing mCherry-Pex15(TA) from plasmid b365 and either (**a**) Sec63p-GFP from plasmid pJK59 or (**b**) sfGFP-ePTS1 expressed from plasmid b311 were examined by fluorescence microscopy.

Table S1. Materials used in this study. Strains (**a**), plasmids (**b**), oligonucleotides (**c**), and antibodies (**d**) are provided, along with construction details.

REFERENCES IN SUPPLEMENTAL TABLE

78–83

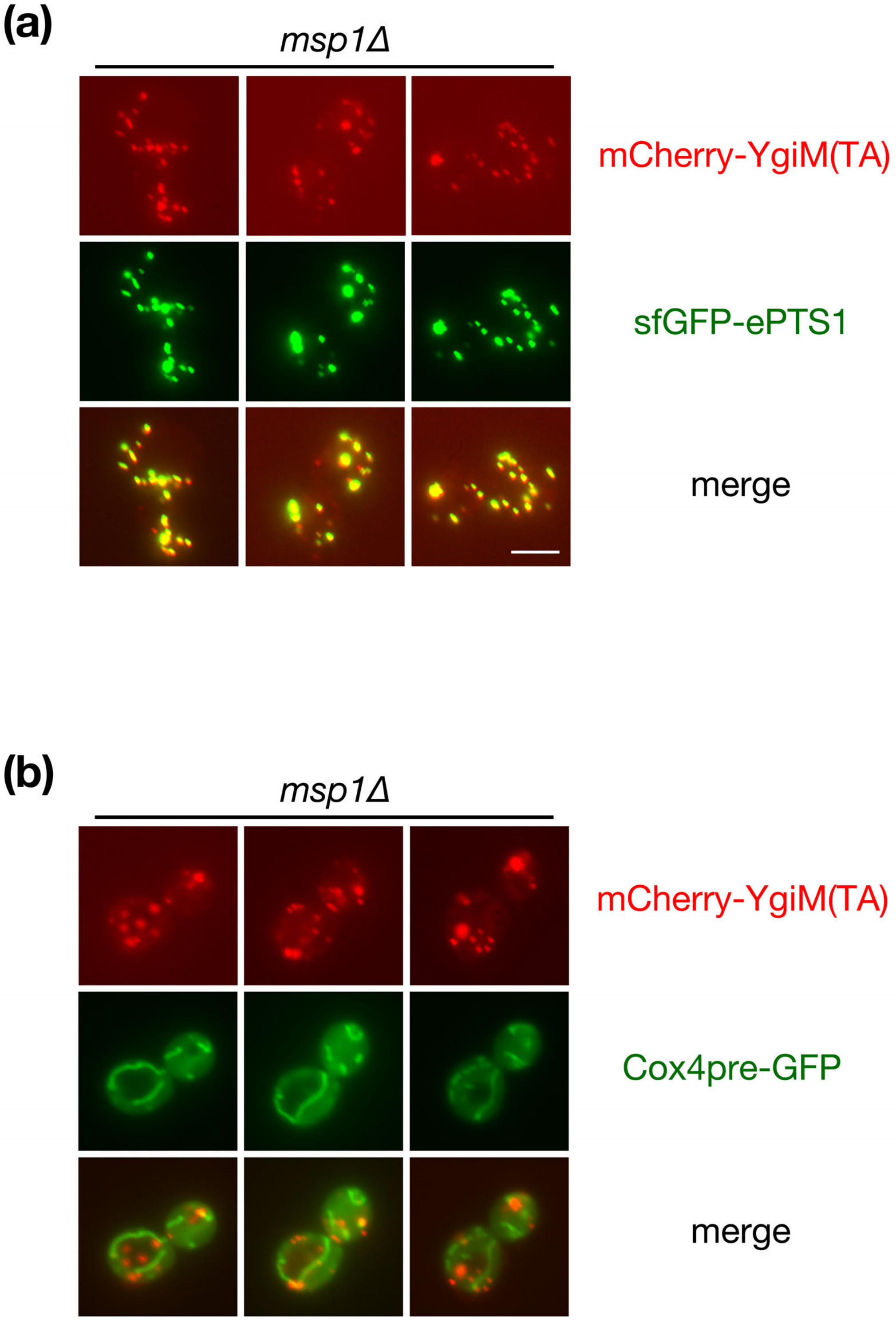
(a)
msp1Δ
mCherry-YgiM(TA)
sfGFP-ePTS1
merge
(b)
msp1Δ
mCherry-YgiM(TA)
Cox4pre-GFP
merge

Figure S1

(a)

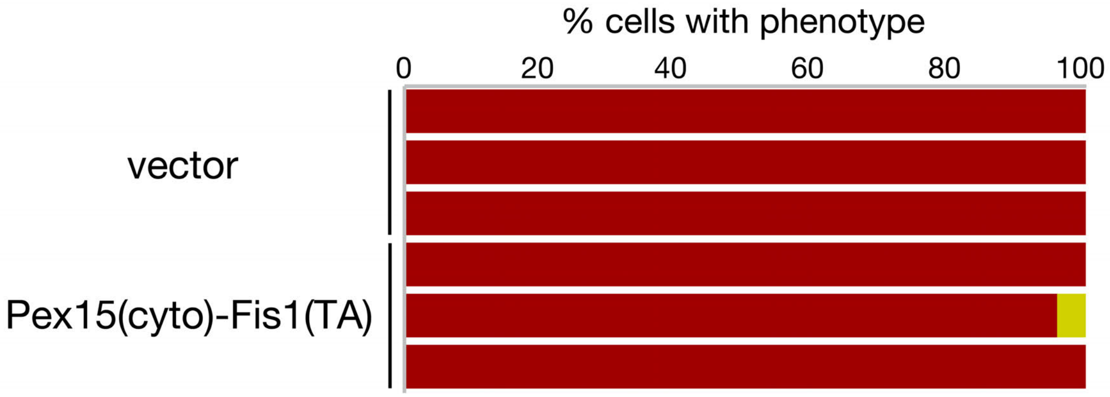
% cells with phenotype
0
20
40
60
80
100
vector
Pex15(cyto)-Fis1(TA)

(b)

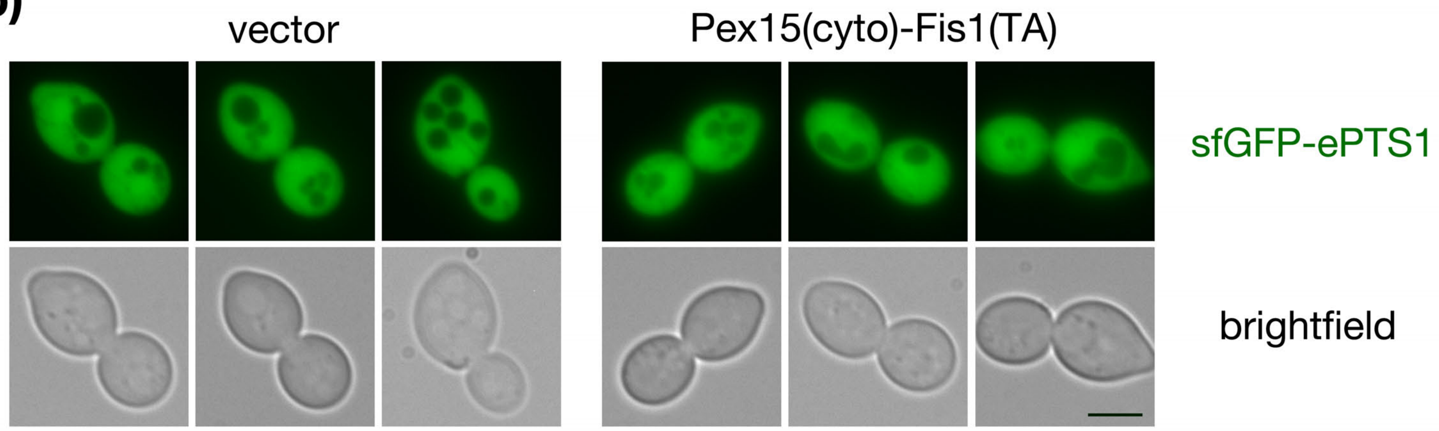
vector
Pex15(cyto)-Fis1(TA)
sfGFP-ePTS1
brightfield

Figure S2

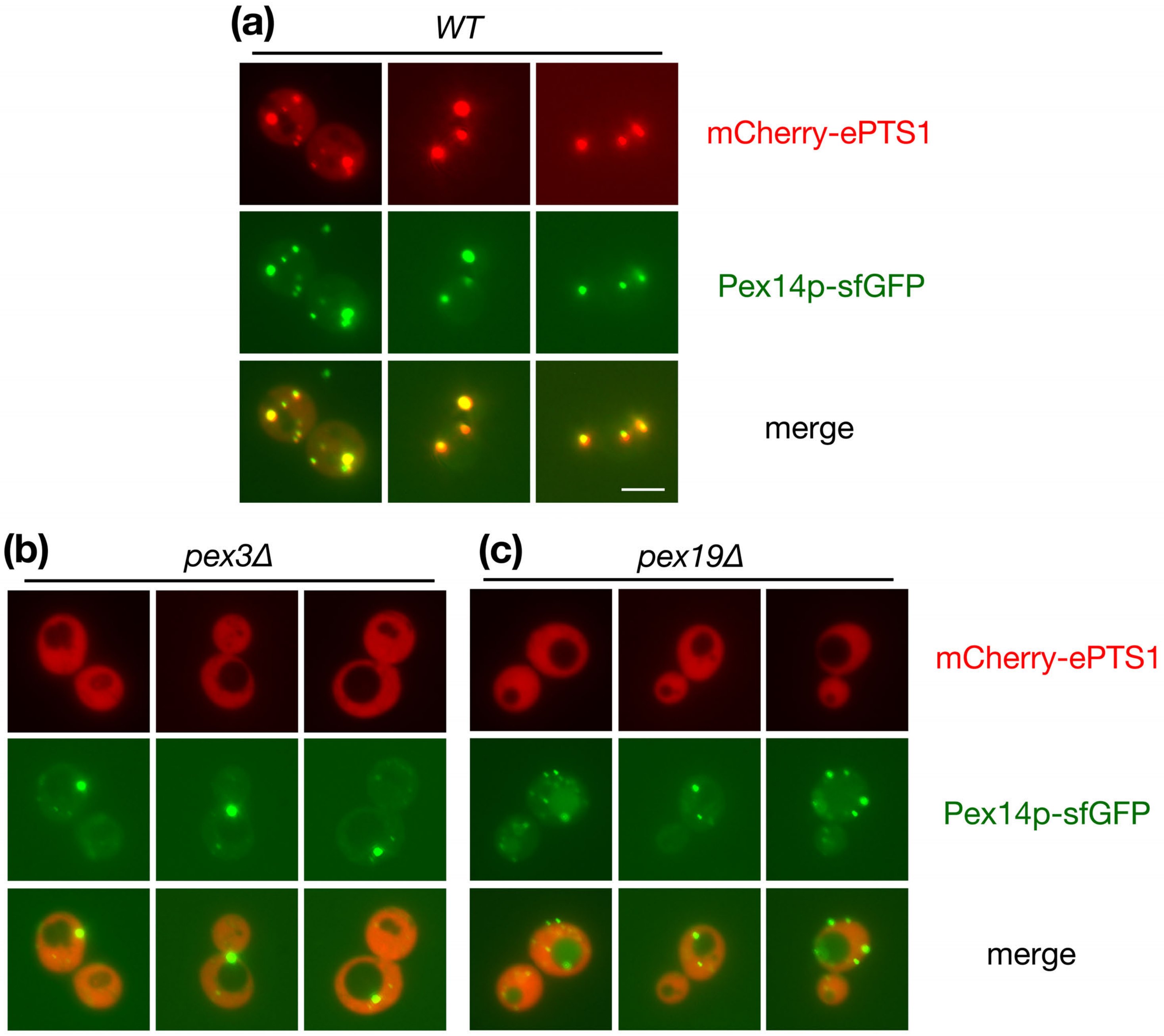
(a)
WT
mCherry-ePTS1
Pex14p-sfGFP
merge
(b)
pex3Δ
(c)
pex19Δ
mCherry-ePTS1
Pex14p-sfGFP
merge

Figure S3

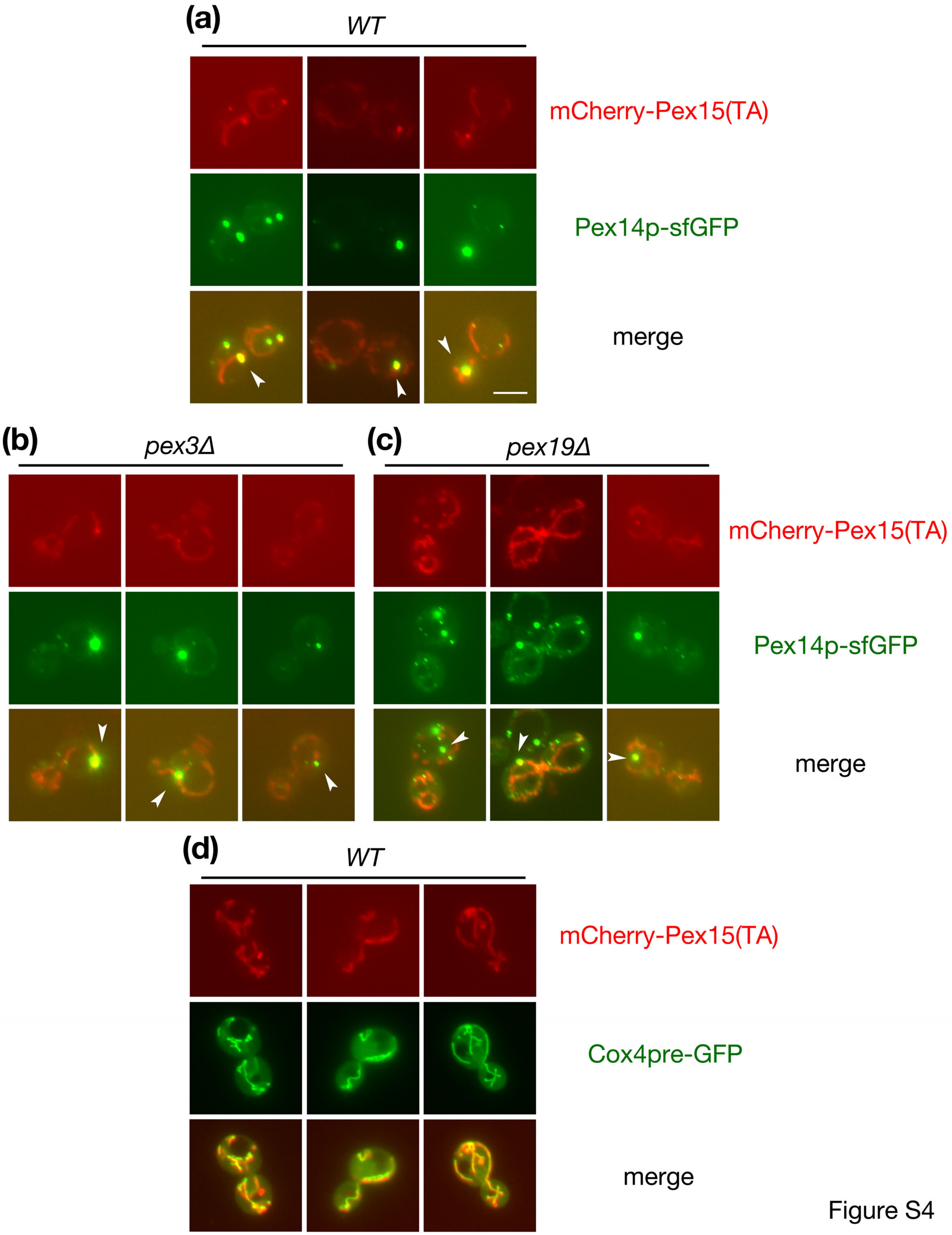
(a)
WT
mCherry-Pex15(TA)
Pex14p-sfGFP
merge
(b)
pex3Δ
(c)
pex19Δ
mCherry-Pex15(TA)
Pex14p-sfGFP
merge
(d)
WT
mCherry-Pex15(TA)
Cox4pre-GFP
merge

Figure S4

(a)

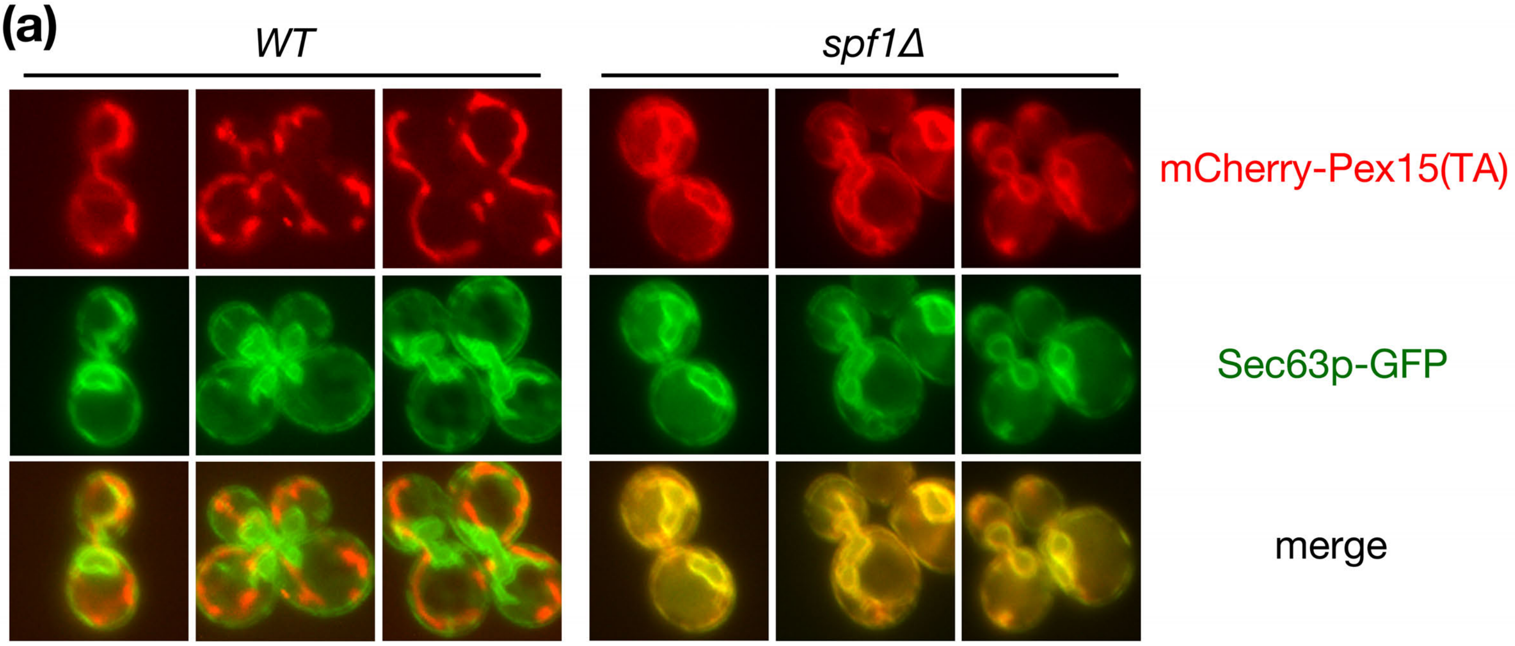
WT
spf1Δ
mCherry-Pex15(TA)
Sec63p-GFP
merge

(b)

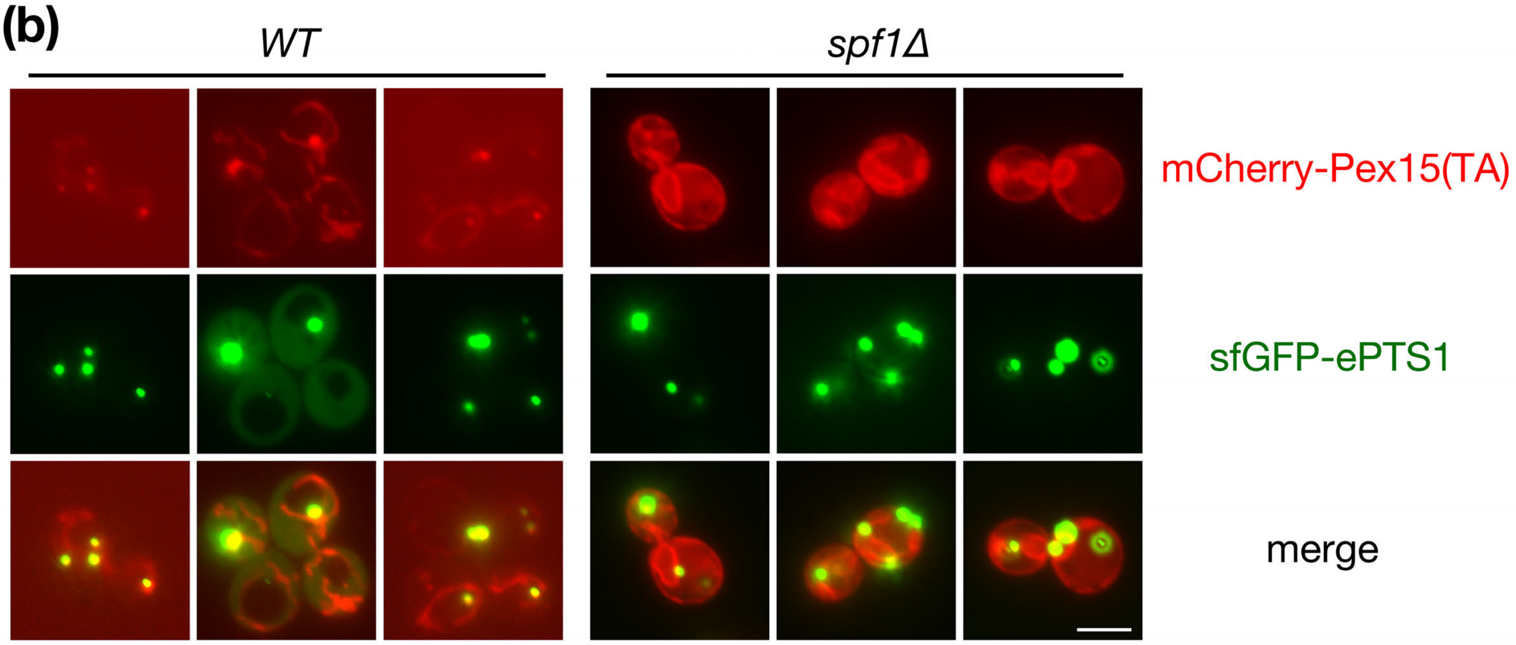
WT
spf1Δ
mCherry-Pex15(TA)
sfGFP-ePTS1
merge

Figure S5
